# Metal–Organic Framework Thin Film-Based Dye Sensitized Solar Cells with Enhanced Photocurrent

**DOI:** 10.3390/ma11101868

**Published:** 2018-10-01

**Authors:** Shargeel Ahmad, Jinxuan Liu, Wei Ji, Licheng Sun

**Affiliations:** 1 State Key Laboratory of Fine Chemicals, Institute of Artificial Photosynthesis, Dalian University of Technology, Dalian 116024, China; shargeel@mail.dlut.edu.cn (S.A.); jiwei@dlut.edu.cn (W.J.); sunlc@dlut.edu.cn (L.S.); 2 Department of Chemistry, School of Chemical Science and Engineering, KTH Royal Institute of Technology, 10044 Stockholm, Sweden

**Keywords:** surface supported metal organic framework, triplet–triplet annihilation upconversion, triplet–triplet energy transfer, dye sensitized solar cell

## Abstract

Metal–organic framework thin film-based dye sensitized solar cell is fabricated with highly oriented, crystalline, and porous Zn-perylene metal-organic framework (MOF) thin film (SURMOF) which is integrated with Bodipy embedded in poly(methyl methacrylate). It has been demonstrated that the photocurrent can be enhanced by a factor of 5 relative to Zn-perylene MOF thin film due to triplet–triplet annihilation up-conversion between the Bodipy/PMMA sensitizer and the Zn-perylene MOF thin film acceptor using Co(bpy)_3_^2+/3+^ as redox mediator.

## 1. Introduction

The fabrication of triplet–triplet annihilation up-conversion (TTA-UC) solar cell device is one of the ongoing interests to overcome the energy loss with suitable energy conversion materials [1,2,3]. The TTA-UC based solar cell devices can overcome the Shockley−Queisser limitations, enhance the efficiency and most importantly decrease the cost [4]. The self-assembled bilayers of sensitizer (S) and acceptor (A) pair on top of metal oxide surface have been reported as one of the promising strategy to enhance the photocurrent via triplet–triplet annihilation (TTA-UC), which can effectively be utilized for dye sensitized solar cells (DSSC) [5]. Surface-anchored metal-organic frameworks (SURMOFs) with controlled growth orientation, regular monolithic porous crystalline frameworks show potential applications in gas separation [6], electronics [7,8,9], CO_2_ reduction [10,11,12,13], water splitting [14,15], photovoltaics [16,17,18], and most recently in TTA-UC system [19,20]. As one of the potential energy materials, the use of SURMOFs for solar energy conversion [16], in particular DSSC, is challenging due to the poor solar capture ability and photo-induced charge carrier mobility, which lead to the sever charge recombination and weak photocurrent. Therefore, it is highly desirable to develop the strategy to enhance the photocurrent in a SURMOF-based DSSC. 

**Figure 1:**
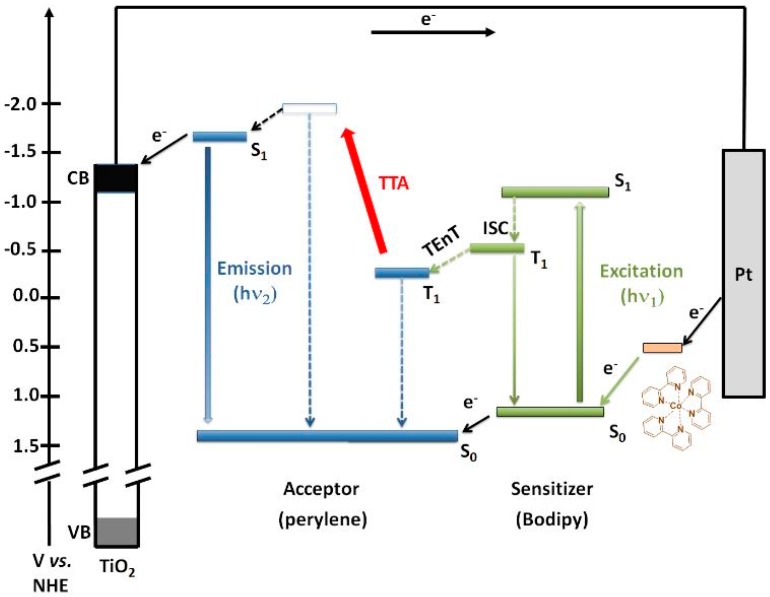
Schematic illustration of energy level diagram of triplet–triplet annihilation up-conversion in DSSC. TEnT: triplet energy transfer.

In TTA-UC process (Figure 1) the photons with low energy (hυ_1_) are absorbed to generate photons of higher energy (hυ_2_) via Dexter type energy transfer mechanism [21] by using low intensity light, followed by energy transfer: (a) first, generation of singlet excited state in sensitizer (S_1_); (b) intersystem crossing (ISC) from singlet into triplet (T_1_); (c) triplet-energy-transfer (TEnT) from the sensitizer to the acceptor; (e) creating the singlet excited state of acceptor through TTA-UC mechanism; (f) finally, the acceptor/emitter emits from its singlet state (Figure 1) [20]. 

An intermediate band solar cell was fabricated in which the conduction band and valance band allow enhanced photovoltaic efficiencies via TTA-UC mechanism [3]. The solar cells based on TTA-UC mechanism have dramatically enhanced photocurrent up to 0.275 mA∙cm^−2^ under 19 equivalent suns [22,23]. Hill et al. has reported a promising material for dye sensitized solar cell (DSSC) with TiO_2_-DPPA-Zn-PtTCPP films (bilayer) (DPPA = 4,4-(anthracene-9,10-diyl)bis(4,1-phenylene) diphosphonic acid, PtTCPP = Pt(II)tetrakis(4-carboxyphenyl)porphyrin) to enhance the photocurrent via TTA-UC [1,24]. 

We have reported previously an epitaxial MOF thin film-based photoelectrochemical device by using highly oriented surface-anchored MOF thin film (SURMOF), Zn-perylene SURMOF as acceptor, and PtOEP (PtOEP = Pt(II) octaethylporphine) as sensitizer in acetonitrile solution [19]. Under green light irradiation, the sensitizer absorbs the photons and transfers the triplet energy to perylene molecules within SURMOFs, followed by the TTA-UC, leading to the enhancement of photocurrent. However, the sensitizers are dissolved in electrolyte solution and randomly contacted with perylene molecules of Zn-perylene SURMOF thin film surface. Therefore, the quenching of oxygen and high local mobility of the sensitizers during TTA-UC process hinders the photocurrent generation efficiency. On the other hand, the metalloporphyrins used as sensitizers for realization of TTA-UC increase the cost of SURMOF-based photovoltaic devices. 

Herein, for the first time, we report an approach by assembling metal-free sensitizer, Bodipy derivative with poly(methyl methacrylate) (PMMA) [25,26,27] on –COOH exposed Zn-perylene SURMOF surface. Recently, the promising idea to use the polymers for the TTA-UC process has been reported [28]. The synthesis and characterizations of the Bodipy derivative (Figures S1 and S2) can be found in Supporting Information. 

**Figure 2:**
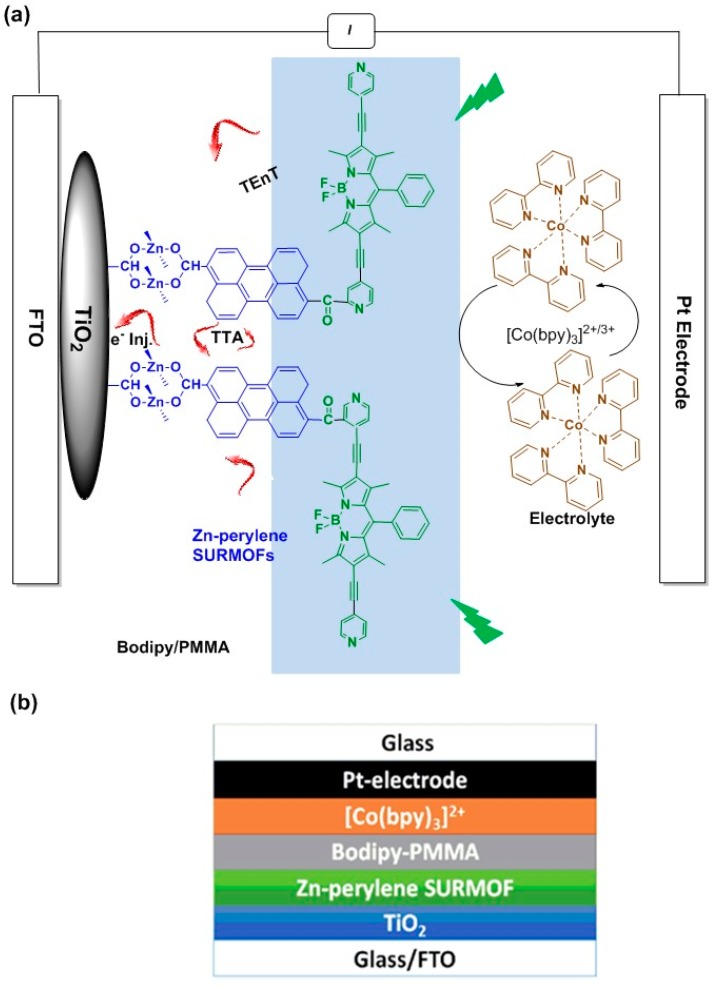
(**a**) Schematic representation of Zn-perylene SURMOF on TiO_2_ and Bodipy/PMMA layer; (**b**) the architecture of Zn-perylene SURMOF-based dye sensitized solar cell.

Further, we assembled this material into a dye-sensitized solar cell (DSSC) and demonstrated the enhancement of photocurrent via TTA-UC in the presence of Co (bpy)_3_^2+/3+^ redox mediator as illustrated in Figure 2. 

The highly crystalline and well oriented Zn-perylene SURMOF with a thickness of ~600 nm was prepared on top of TiO_2_ according to our previously reported protocol [19]. In the next step, the –COOH exposed Zn-perylene SURMOF was modified with Bodipy sensitizers [29] and PMMA via photochemical reaction which formed a glassy film of Bodipy/PMMA on top of TiO_2_-Zn-perylene SURMOF (Figure S3). The detailed fabrication procedure can be found in Supporting Information.

## 2. Results and Discussions

**Figure 3:**
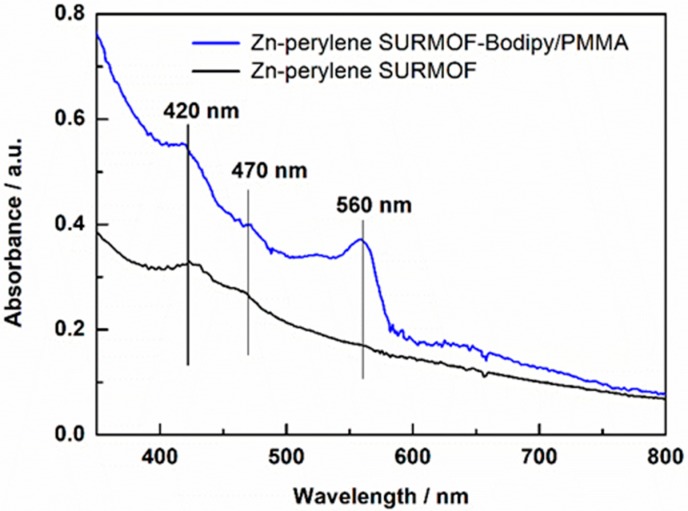
UV–vis spectra of Zn-perylene SURMOF-Bodipy/PMMA (in blue) and Zn-perylene SURMOF (in black).

The ultraviolet-visible (UV–vis) spectrum of Zn-perylene SURMOF is shown in Figure 3 (in black). Compared with the UV–vis spectrum of free perylene dicarboxylic acids (438 nm and 460 nm in acetonitrile solution, Figure S4), Zn-perylene SURMOF shows absorption band at 420 nm and 470 nm, which are associated to the S_1_ (B_1u_) ← S_0_ (A_g_) transition of perylene units [30]. After integration of as prepared SURMOF with Bodipy/PMMA, a new absorption band at 560 nm was observed, which can be attributed to the π–π* transition of Bodipy compounds [31].

The fabricated hybrid material was further characterized by infrared (IR) spectroscopy as shown in Figure S5. The Zn-perylene SURMOF-Bodipy/PMMA material showed two strong bands appeared at 1746 cm^−1^ and 1725 cm^−1^ which can be attributed to the carbonyl group of PMMA [32]. Due to the small amount of Bodipy in PMMA, the vibrational bands of Bodipy are overlapped with the bands of PMMA and Zn-perylene SURMOF (Table S1).

A SURMOF-based DSSC device was fabricated according to previously reported protocol [16] as shown in Figure 2b. We rationalized to utilize the new materials of Zn-perylene SURMOF-Bodipy/PMMA/TiO_2_ as photoanode, platinum as counter electrode and Co (bpy)_3_^2+/3+^ as redox mediator in acetonitrile solution. The detailed fabrication procedure can be found in Supporting Information. 

The chronoamperometric (i-t) experiments were performed by using TiO_2_-Zn-perylene SURMOF-Bodipy/PMMA, TiO_2_-Zn-perylene SURMOF and TiO_2_ as photo anode and platinum as counter electrode in Co (bpy)_3_^2+/3+^ acetonitrile solution with an external potential 0 V vs Ag/AgNO_3_. A 532 nm green light (power: ~80 mW∙cm^−2^) coupled with an automatic shutter was used to control the light irradiation—i.e., light on and light off. 

**Figure 4:**
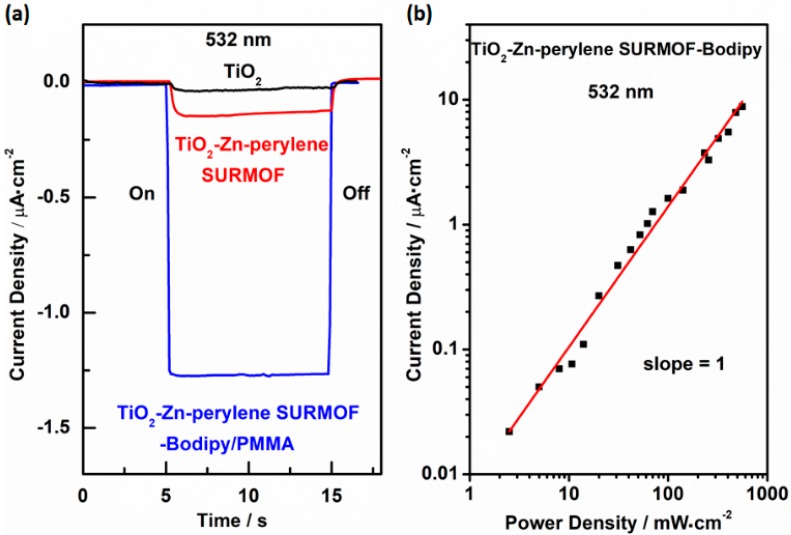
(**a**) The i-t curves for DSSCs composed of TiO_2_-Zn-perylene SURMOF-Bodipy/PMMA, TiO_2_-Zn-perylene SURMOF, and TiO_2_ as photoanodes and Co(bpy)_3_^2+/3+^ as redox mediator under the 532 nm light irradiation (power: ~80 mW∙cm^−2^). (**b**) The current density of TiO_2_-Zn-perylene SURMOF-Bodipy/PMMA under 532 nm green light irradiation with different power intensity.

As shown in Figure 4a and Figure S6, under the 532 nm light irradiation, the transient photocurrents of ~1.25 μA∙cm^−2^, ~0.25 μA∙cm^−2^, and ~0.1 μA∙cm^−2^ are obtained for TiO_2_-Zn-perylene SURMOF-Bodipy/PMMA, TiO_2_-Zn-perylene SURMOF and TiO_2_, respectively. The photocurrent was enhanced nearly 5-fold compared to that of TiO_2_-Zn-perylene SURMOF and TiO_2_. 

It has been reported that the distance between photosensitizer and acceptor should be between agreeable levels to enhance the energy via TTA-UC [2]. The addition of solidified PMMA with Bodipy on top of –COOH exposed Zn-perylene MOF thin film has considerably contributed for the energy conversion performance of the materials [33]. As shown in Figure S13 that TiO_2_-Zn-perylene SURMOF + Bodipy/PMMA has greater solar energy conversion performance than TiO_2_-Zn-perylne SURMOF, TiO_2_-Bodipy/PMMA, and TiO_2_-electrolyte. Therefore, the strategy to utilize novel Zn-perylene SURMOF-Bodipy/PMMA materials shows potential applications for DSSC. 

In order to have a deep understanding about the mechanism of photocurrent enhancement, we have carried out fluorescence experiment by using perylene dicarboxylic acid as acceptor and Bodipy as sensitizer in acetonitrile solution as shown in Figure S7. Upon excitation wavelength of 532 nm for the perylene dicarboxylic acid + Bodipy system the emission signal at 496 nm and 582 nm was observed (Figure S7 inlet) which can be attributed to emission signal from perylene dicarboxylic acid and Bodipy emission. As a reference the emission spectra of biphenyl dicarboxylic acid + Bodipy in deaerated acetonitrile was displayed in Figure S8, which gives no emission signal indicating that the TTA-UC cannot occur between biphenyl unit and Bodipy. Based on the above-mentioned results, we have proposed the TTA-UC mechanism to explain the photocurrent enhancement for Zn-perylene SURMOF + Bodipy system. Upon 532 nm excitation, the triplet energy generated from Bodipy sensitizer with higher energy and long-lived life time allows them to efficiently transfer triplet energy (via triplet energy transfer (TEnT)) to the Zn-perylene SURMOF acceptor. The perylene molecules annihilate the triplet energy via TTA-UC to generate the excited singlets which diffuse through the Zn-perylene SURMOF to the interface between Zn-perylene SURMOF and TiO_2_, where the charge separation occurs and the generated electrons are injected into the TiO_2_ for photocurrent enhancement (Figure 2a). 

In addition, the power intensity dependent current density experiment which is one of the standard measurements for support of TTA-UC mechanism was carried out as displayed in Figure 4b. Upon 532 nm green light excitation, a linear response was obtained from all power range which is different from quartic to linear behavior observed in TiO_2_-Zn-perylene SURMOF-PtOEP (PtOEP = Pt(II) octaethylporphine) [19] due to the efficient TEnT process [30], which favors the up-conversion process with a linear relationship than a quadratic relationship [27,34]. 

The Bodipy sensitizers encapsulated in solid-state-like PMMA film material can overcome the non-radiative decay process and assist the efficient TEnT from Bodipy to Zn-perylene SURMOF (acceptor) as schematically shown in Figure 1. In such a system, the generated triplets from photosensitizers have enough time to transfer triplet energy more efficiently to the acceptor and go through TTA-UC to produce photocurrent with TiO_2_-Zn-perylene SURMOF-Bodipy/PMMA device (Figure 2a). 

It is inferred that the quenching effect in TiO_2_-Zn-perylene SURMOFs-Bodipy/PMMA materials based device can be suppressed, where the generated triplet energy from Bodipy sensitizer can be efficiently transferred to the acceptors and further generate enhanced photocurrent via TTA-UC [24]. 

Nanosecond transient absorption spectroscopy (TAS) measurement has been performed to characterize the triplet lifetime of Bodipy in acetonitrile with and without the presence of O_2_ as shown in Figures S9 and S10. In deaerated acetonitrile, the generated triplet lifetime (τ) with a value of 240 μs was obtained, while the transient was significantly quenched in aerated acetonitrile (τ = 280 ns). Furthermore, we have performed another control experiment and found that in the presence of oxygen the photocurrent cannot be enhanced for TiO_2_-Zn-perylene SURMOF-Bodipy (without PMMA) due to the quenching of O_2_ (Figure S11).

Moreover, the chronoamperometric experiments were also performed by using a 430 nm blue laser (power ≈ 60 mW∙cm^−2^). As shown in Figure S12, the transient photocurrent of ~10 μA∙cm^−2^ for TiO_2_-Zn-perylene SURMOF-Bodipy/PMMA materials-based devices was nearly 2-fold higher than that of (~4.5 μA∙cm^−2^) for TiO_2_-Zn-perylene SURMOF and ~1.2 μA∙cm^−2^ for TiO_2_. The TiO_2_-Zn-perylene SURMOF-Bodipy/PMMA device exhibited a linear response upon 430 nm blue light irradiation at low power intensity due to the faster triplet energy transfer from Bodipy to Zn-perylene SURMOF [23].

The overall photovoltaic performances of TiO_2_-Zn-perylene SURMOF-Bodipy/PMMA device were examined as shown in Figure S13 and Table S2. The device using TiO_2_-Zn-perylene SURMOF-Bodipy/PMMA as photoanode exhibits the best performances among the devices using TiO_2_-Zn-perylene SURMOF, TiO_2_-Bodipy/PMMA, and TiO_2_ as photoanode due to TTA-UC [19,20]. Although the device performance is not very impressive, in the present work, we demonstrated the strategy with the upconverting SURMOF-based system for solar energy conversion. It requires a lot of study to further develop the MOF thin film materials for PV applications. Further research efforts on the improvement of the device performances have been planned and will be carried out in the future.

## 3. Conclusions

In conclusion, we have fabricated a prototype Zn-perylene SURMOF material based TTA-UC device which is integrated with metal-free Bodipy sensitizer and glassy PMMA polymer. It has been demonstrated that the photocurrent can considerably be enhanced via TTA UC due the Zn-perylene SURMOF-Bodipy/PMMA materials-based devices. However, it also suggests a need for more extensive research efforts towards study of detailed mechanisms, exploration of suitable electrolytes, and optimization procedures for device based on these materials. The proposed investigation will further improve the potential for enhancing the efficiency of MOF thin film materials-based energy conversion devices. Thus, the dedicated efforts in such direction may raise the interest for solidified smart and hybrid materials based solar energy conversion devices.

## Supplementary Materials

The following are available online at http://www.mdpi.com/1996-1944/11/10/1868/s1.
