# Supplementary material for: Metal–Organic Framework Thin Film-Based Dye Sensitized Solar Cells with Enhanced Photocurrent
Source: Materials (Basel). 2018 Oct 1;11(10):1868. doi: 10.3390/ma11101868 (PMC6213061; doi:10.3390/ma11101868)
Supplement: Supplementary file 1 [file materials-11-01868-s001.pdf]

materials

Supporting Information

# Metal-Organic Framework Thin Film-based Dye Sensitized Solar Cells with Enhanced Photocurrent

**Shargeel Ahmad [1], Jinxuan Liu [1,*], Wei Ji [1], and Licheng Sun [1,2]**

[1] State Key Laboratory of Fine Chemicals, Institute of Artificial Photosynthesis, Dalian University of Technology, Dalian 116024, China; shargeel@mail.dlut.edu.cn (S.A.); jiwei@dlut.edu.cn (W.J.); sunlc@dlut.edu.cn (L.S.)
[2] Department of Chemistry, School of Chemical Science and Engineering, KTH Royal Institute of Technology, 10044 Stockholm, Sweden
* Correspondence: jinxuan.liu@dlut.edu.cn

Received: 5 September 2018; Accepted: 28 September 2018; Published: 1 October 2018

## 1. Materials and Methods

### 1.1. Materials

All the chemicals were purchased from Sigma Aldrich. The TiO2 18-NRT is purchased from Heptachroma Company. All the used chemicals are analytically pure and used as received.

### 1.2. Methods

The NMR spectra were recorded with OXFPRD NMR 400 MHz spectrometer using CDCl3 as solvent and tetramethylsilane (TMS) as standard at 0.00 ppm. HRMS was accomplished with matrix-assisted laser desorption/ ionization (MALDI) micro MALDI TOF mass spectrometer (Waters, U.K.). The Nanosecond transient absorption spectroscopy was measured with a LP920 laser flash photolysis spectrometer (Edinburgh Instruments, Livingston, U.K.). Firstly, the samples were purged with N2 for 15 min before measurements, and the N2 gas flow was kept constant during the measurement. The signal was digitized with a Tektronix TDS 3012B oscilloscope. The UV-Vis measurement was recorded with Agilent 8453 instrument. The fluorescence spectra of Zn-perylene SURMOFs were measured with a spectroflourometer (Flouromax-4 spectroflourometer-Horiba. Laser sources for our experiments are purchased from Changchun New Industries ptoelectronics Technology Co., Ltd. Green Laser). The infrared spectra of the SURMOF sample were obtained with a resolution of 2 cm-1 with FTIR spectrometer (Bruker VERTEX 80v) under vacuum (~3 mbar) equipped with a liquid nitrogen cooled narrow band mercury cadmium telluride (MCT) detector. The 3,9-perylenedicarboxylic acid reference samples were prepared into a KBr pellet and measured in transmission mode. The i–t curves were recorded with a potentiostat (CHI 660D, Shanghai, China) using diode pumped solid state (DPSS) continuous laser (530 nm, 430 nm) (Changchun New Industries Optoelectronics Technology Co., Ltd. Green Laser). The dye sensitized solar cells having two electrode system consisting of SURMOFs sample as working electode and Pt-electrode as counter electrode were used. The SHIMAZDU spectrofluorophotometer (RF-5301pc) with laser sources was used for the upconversion. The power of the laser beam was measured with VLP-2000 pyroelectric laser power meter.

## 2. Synthesis of Bodipy derivative

The Bodipy derivative 3 was synthesized according to the scheme as shown in Scheme 1.

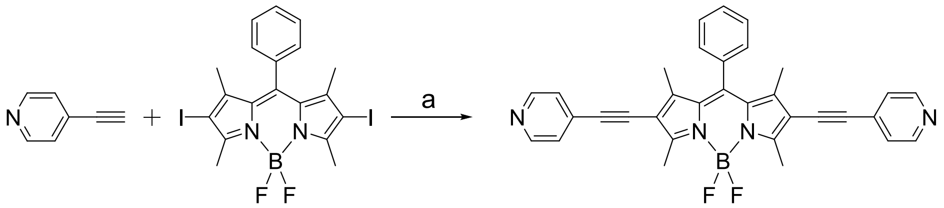


1 2 3

**Scheme 1.** The synthetic route of Bodipy derivative 3. a $Pd(PPh_3)_2Cl_2$, $PPh_3$, CuI, distilled THF, dried $NEt_3$, $N_2$, 50 °C, 12 h, yield: 72%.

**1** was synthesized according to literature methods[1].

**2** was synthesized according to literature methods[2].

Synthesis of **3**. **1** (210 mg, 2 mmol) and **2** (170 mg, 0.3 mmol) was dissolved in the mixture of distilled THF (12 mL) and dried $Et_3N$ (3 mL). $Pd(PPh_3)_2Cl_2$ (10 mg, 0.015 mmol) and $PPh_3$ (4 mg, 0.015 mmol) were added under $N_2$, followed by CuI (6 mg, 0.03 mmol). The mixture was stirred under $N_2$ for 12 h at 50 °C. After the reaction was finished, the solvent was removed under reduced pressure. The residue was purified by column chromatography (silica gel, $CH_2Cl_2$/MeOH = 100:1, v/v). **3** was collected as a dark red solid (133 mg, yield: 72%). $^1$H NMR (400 MHz, $CDCl_3$): $\delta$ 8.57 (d, 4H, $J$ = 5.7 Hz), 7.58–7.55 (m, 3H), 7.32–7.29 (m, 6H), 2.73 (s, 6H), 1.54 (s, 6H). MALDI-TOF-HRMS ($[C_{33}H_{25}BN_4F_2 + H]^+$): calcd m/z = 527.2219, found m/z = 527.2236. Ethanol and acetonitrile were distilled before use and kept under argon atmosphere.

## 3. Preparation of Zn-Perylene SURMOF

The FTO/$TiO_2$ substrates are used for the preparation of the Zn-Perylene MOFs thin film with liquid-phase epitaxy. Firstly, a concentration of 1 mM ethanolic solution of zinc acetate (spray time: 15 s, waiting time: 30 s) was sprayed on FTO-$TiO_2$ substrate. Secondly, ethanolic solution of 40 μM perylene dicarboxylic acid (spray time: 20 s, waiting time: 30 s) were deposited on the first layer of zinc acetate. The first cycle of Zn acetate supports the formation of MOF thin film[3]. Similarly, all cycles were layer by layer deposited onto FTO/$TiO_2$ substrates at room temperature with humidity of 25–35%.

## 4. Preparation of FTO/TiO2-Zn-Perylene SURMOF-Bodipy/PMMA

First, 40 mg/mL PMMA was prepared in the acetonitrile solution. Subsequently, pyridine functionalized Bodipy with a concentration of 30 M was added into the aforementioned PMMA solution. Then, the FTO-$TiO_2$-Zn-perylene SURMOF was immersed into the mixture of PMMA and Bodipy solution for one hour. During immersion, the solution was bubbled with $N_2$ under light irradiation. Finally, after drying with $N_2$, the Bodipy/PMMA layer was formed on top of FTO/$TiO_2$-Zn-perylene SURMOF as shown in Figure S2. The thickness of Bodipy/PMMA is dependent on the color change on the surface of Zn-perylene SURMOF which is shown in Figure S3.

## 5. Fabrication of DSSC-like device with Zn-perylene SURMOF

The DSSC-like devices with Zn-perylene SURMOF were prepared according to the previous literature method[4] using $Co(bpy)_3(PF_6)_2$ (0.22 M) as electrolyte instead of $I^-/I_3^-$.

## 6. Photoelectrochemical characterization

The i–t curves were recorded with a potentiostat using the DSSC-like device using FTO-$TiO_2$-Zn-perylene SURMOF-Bodipy/PMMA, FTO-$TiO_2$-Zn-perylene SURMOF and FTO-$TiO_2$-Bodipy/PMMA as working electrode, Pt as counter electrode and $Co(bpy)_3^{2+/3+}$ in

acetonitrile as electrolyte. A 530 nm green laser and a 430 nm green laser were used for light irradiations

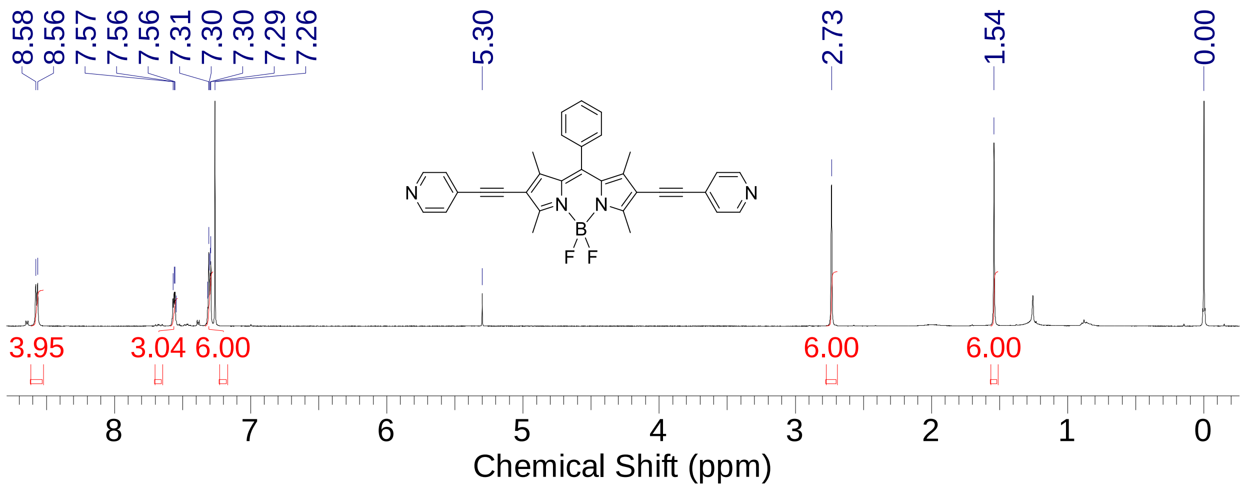


**Figure S1.** 1H NMR spectrum of **3** (400 MHz, $CDCl_3$), 25 °C.

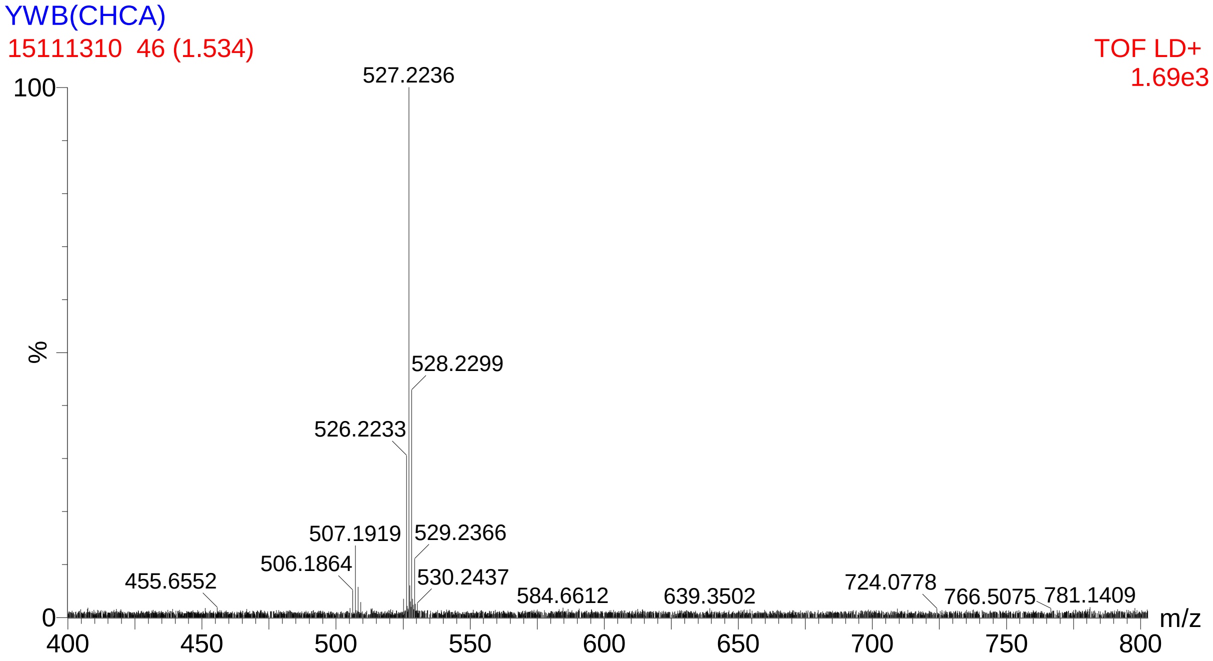


**Figure S2.** MALDI–TOF–HRMS spectrum of **3**.

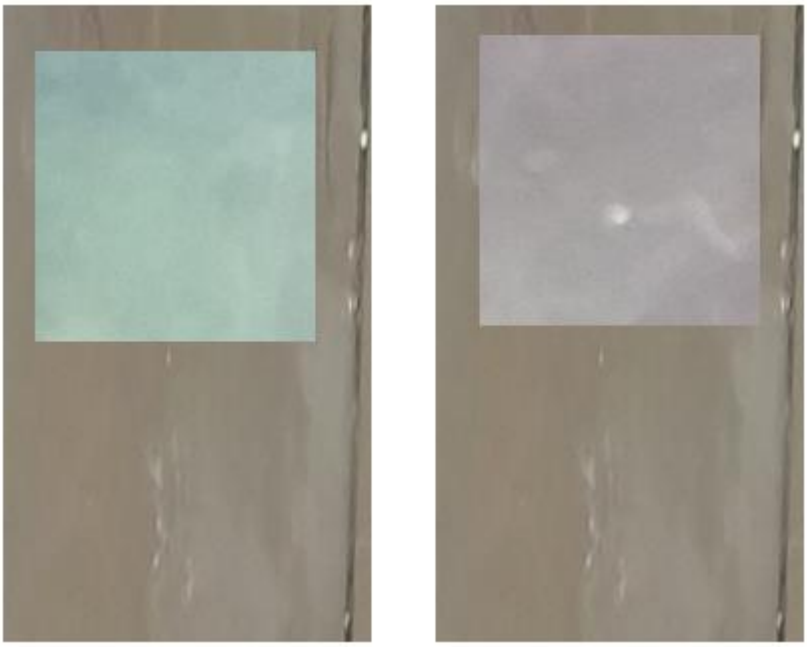

**Figure S3.** Optical images of (left) FTO/$TiO_2$-Zn-perylene SURMOF and (right) FTO/$TiO_2$-Zn-perylene SURMOF-Bodipy/PMMA.

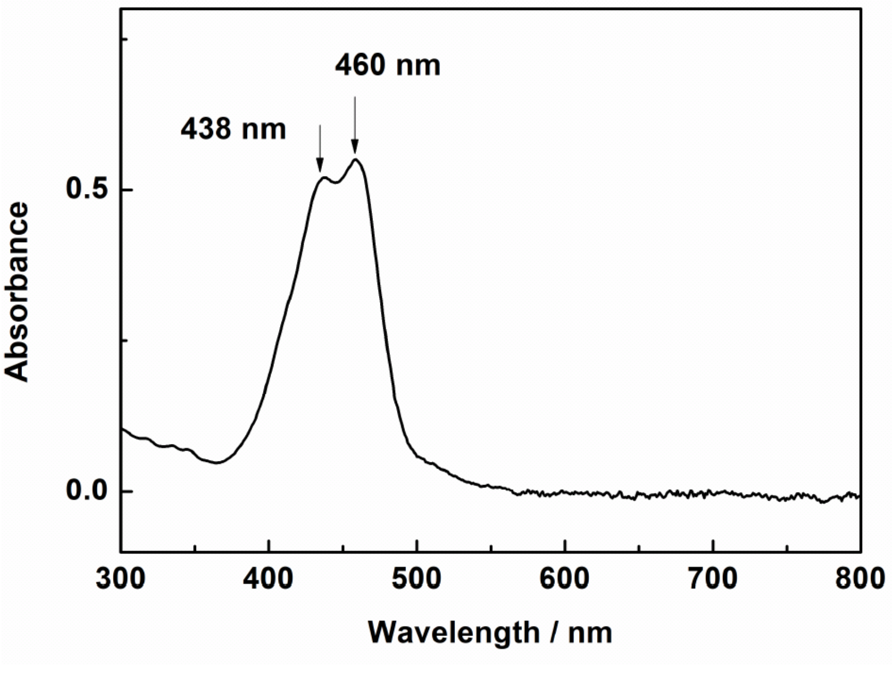


**Figure S4.** UV-vis spectrum of perylene dicarboxylic acid solution.

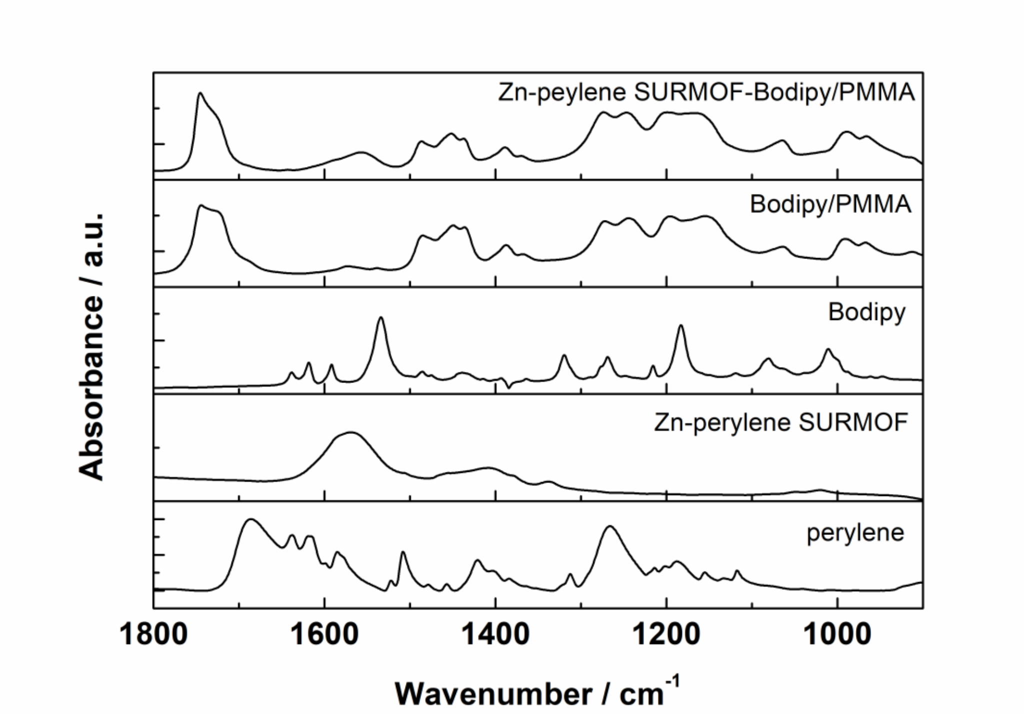


**Figure S5.** Infrared spectra of Zn-perylene SURMOF-Bodipy/PMMA, Bodipy/PMMA, Bodipy powder, Zn-perylene SURMOFs and perylene powder.

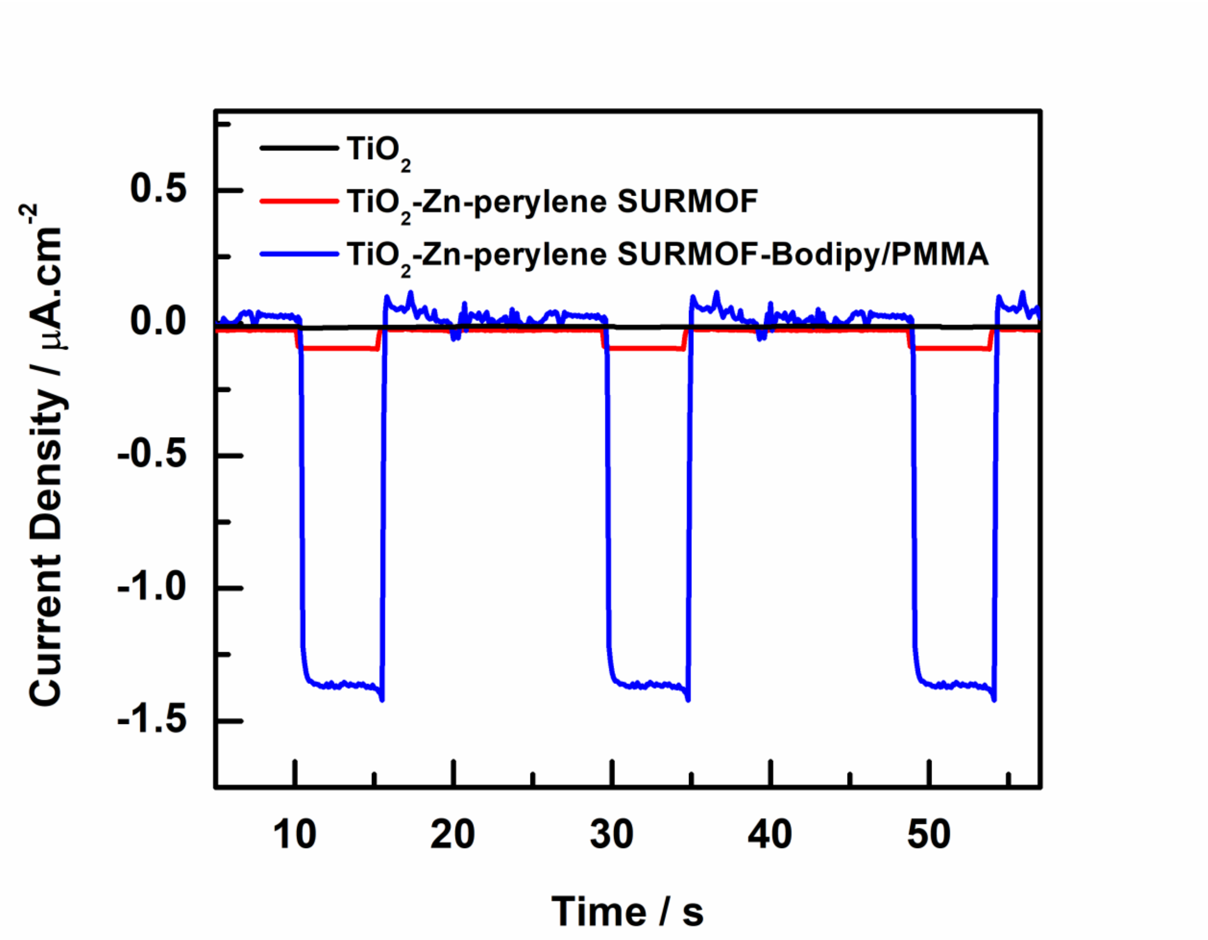


**Figure S6.** (**a**) The i-t curves (3 cycles) for DSSCs composed of $TiO_2$-Zn-perylene SURMOF-Bodipy/PMMA, $TiO_2$-Zn-perylene SURMOF, and $TiO_2$ as photoanodes and $Co(bpy)_3^{2+/3+}$ as redox mediator under the 532 nm light irradiation (power: ~ 80 $mW \cdot cm^{-2}$).

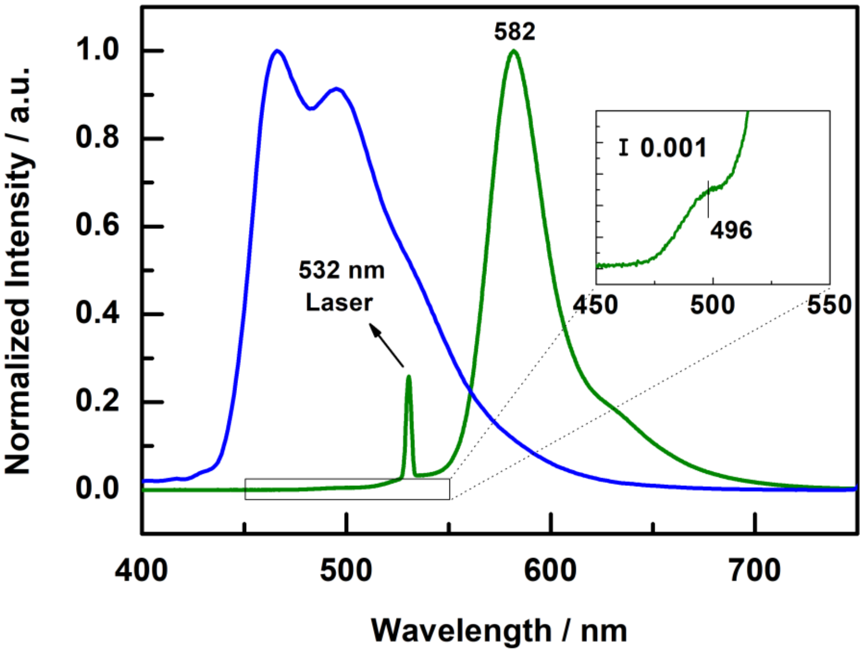


**Figure S7.** Emission spectra of perylene dicarboxylic acid (blue, $\lambda$ex = 430 nm) and perylene dicarboxylic acid + Bodipy (green, $\lambda$ex = 532 nm) in deaerated acetonitrile solution. The 532-nm excitation peak is attributed to the wavelength of laser source. Excitation power density of 4.6 mW/cm$^2$ was used to for 532 nm laser source.

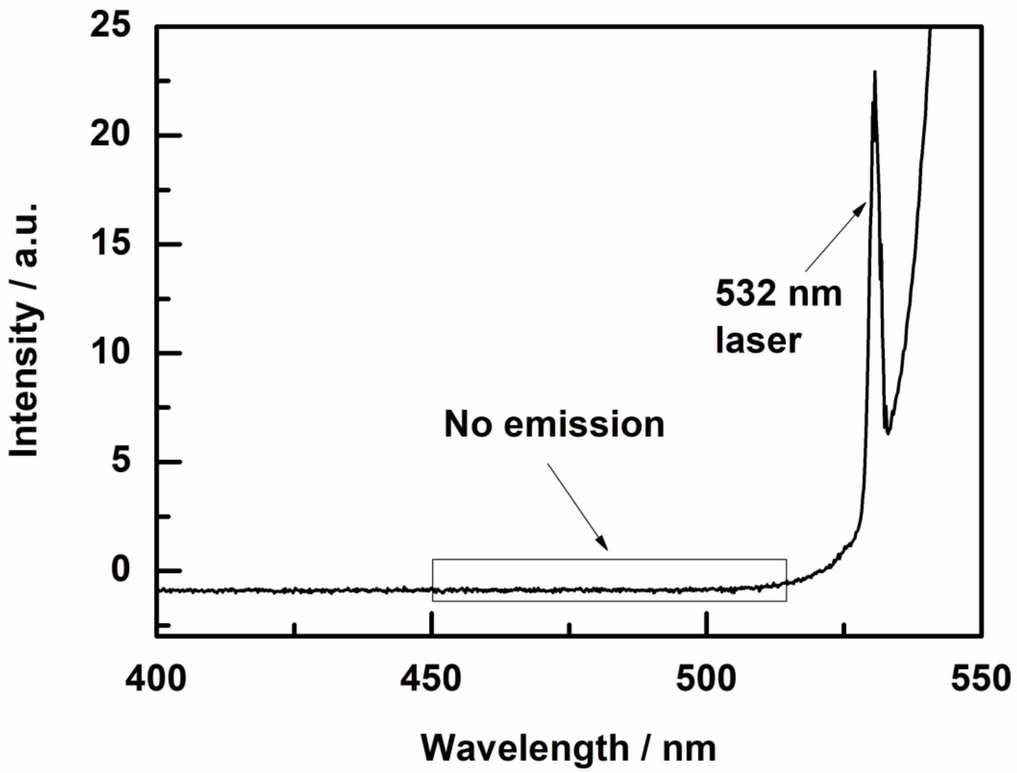


**Figure S8.** Emission spectra of biphenyl dicarboxylic acid + Bodipy (green, $\lambda$ex = 532 nm) in deaerated acetonitrile solution. The 532-nm excitation peak is attributed to the wavelength of laser source. Excitation power density of 4.6 mW/cm$^2$ was used to for 530-nm laser source.

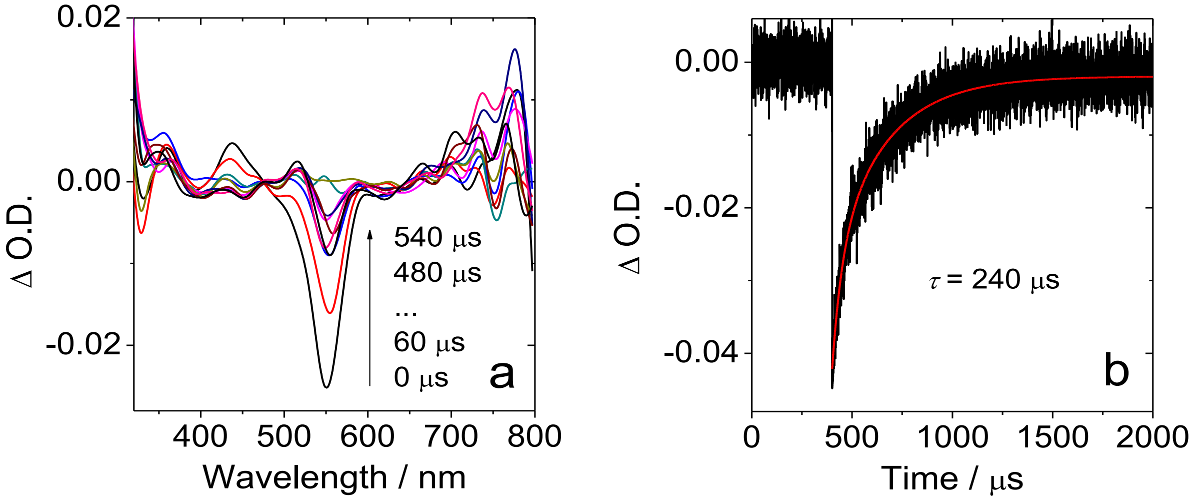


**Figure S9.** (**a**) Nanosecond transient absorption spectra of **3**, (**b**) decay curve at 555 nm. $\lambda_{ex}$ = 532 nm, $c$ = 1.0 × $10^{-5}$ M in deaerated acetonitrile at 20 °C.

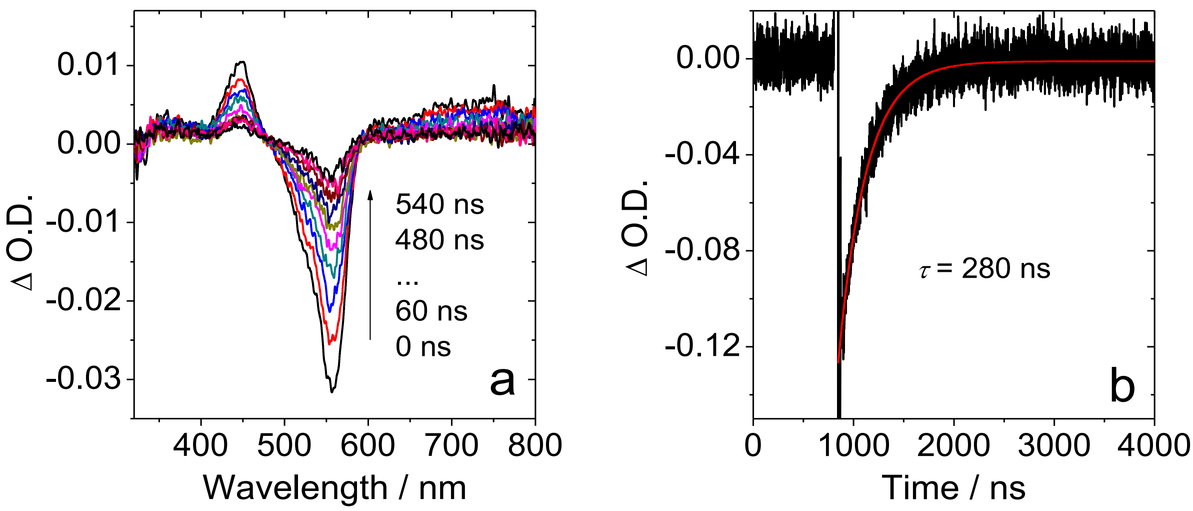


**Figure S10.** (**a**) Nanosecond transient absorption spectra of **3**, (**b**) decay curve at 555 nm. $\lambda_{ex}$ = 532 nm, $c$ = 1.0 × $10^{-5}$ M in aerated acetonitrile at 20 °C.

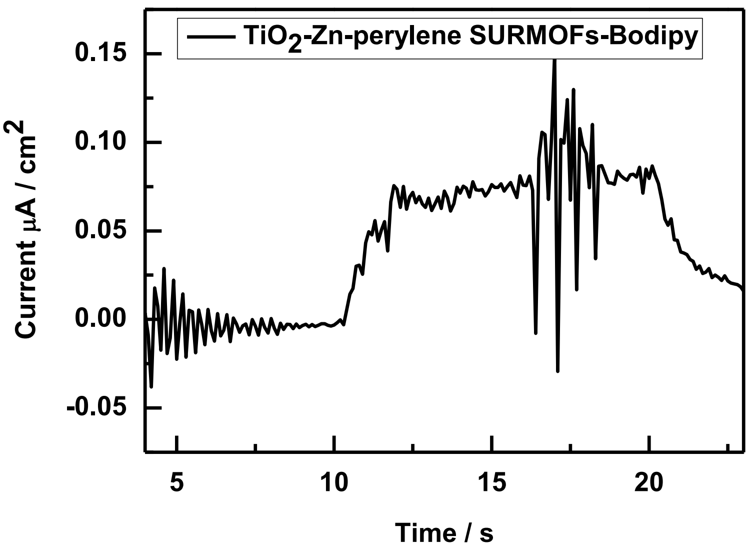


**Figure S11.** The photocurrent response of $TiO_2$-Zn-perylene SURMOFs + Bodipy (without PMMA) in the presence of oxygen with 532 nm green light.

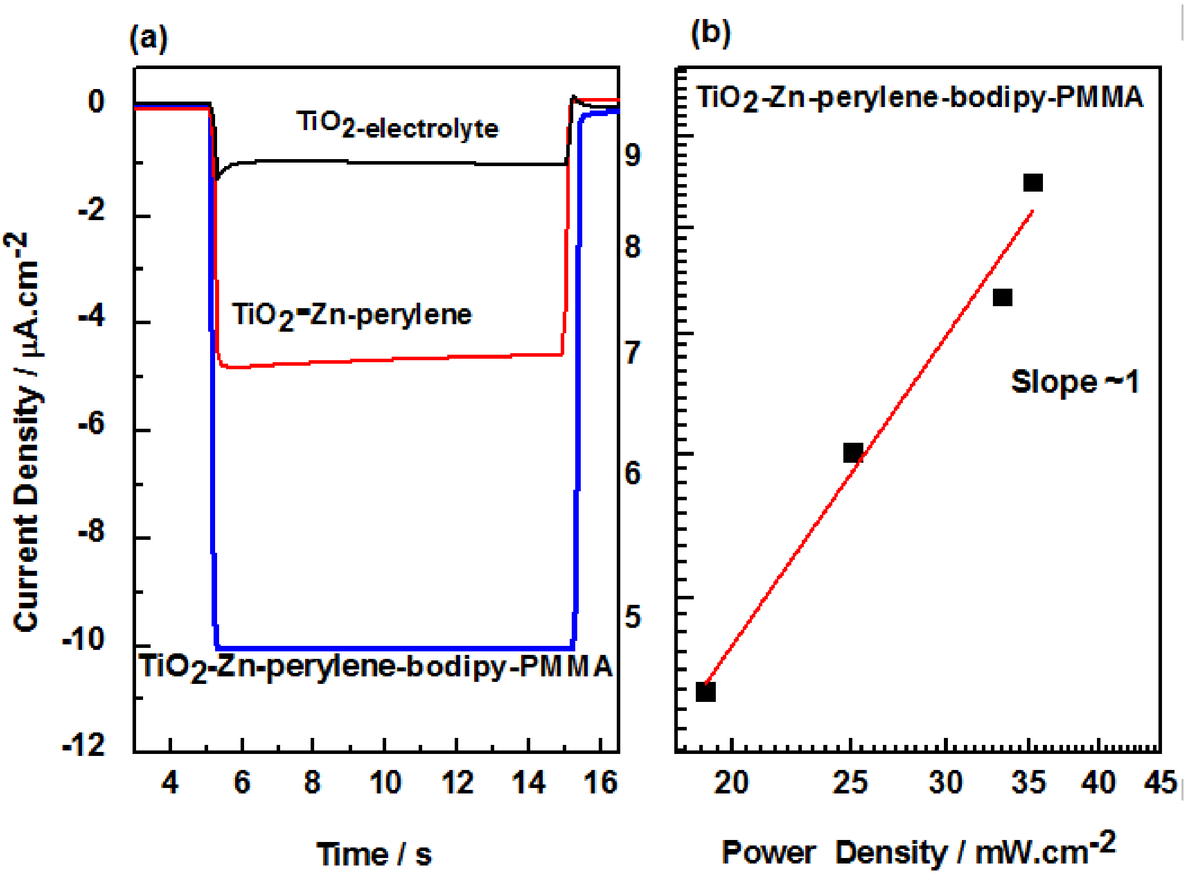


**Figure S12.** (**a**) The i–t curves for DSSCs composed of $TiO_2$-Zn-perylene SURMOF-Bodipy/PMMA, $TiO_2$-Zn-perylene SURMOF, and $TiO_2$ as photoanode and $Co(bpy)_3^{2+/3+}$ as redox mediator; (**b**) The current density of $TiO_2$-Zn-perylene-Bodipy/PMMA under 430 nm light irradiation with various power intensity.

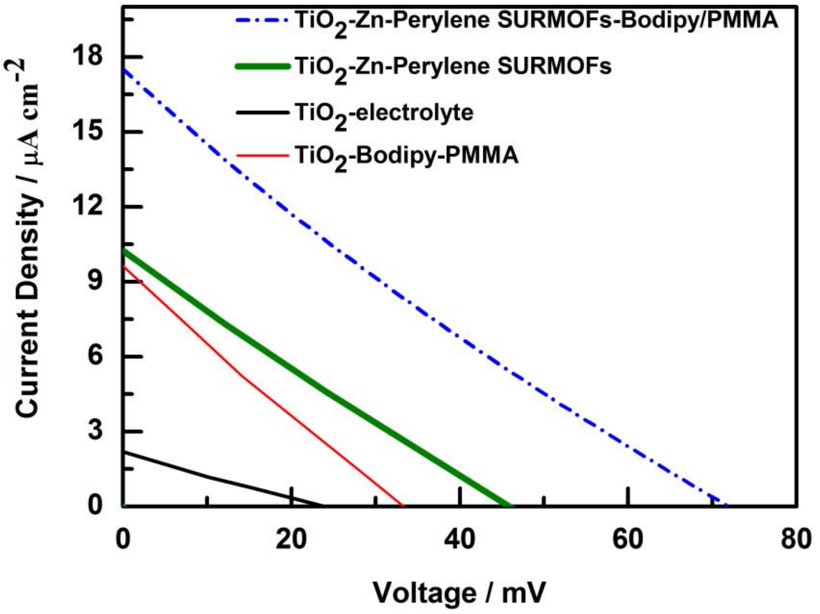


**Figure S13.** Photocurrent versus voltage *(J–V)* characteristics for $TiO_2$-Zn-perylene SURMOFs-Bodipy/PMMA (blue), $TiO_2$-Zn-perylene SURMOFs (green) and $TiO_2$-electrolyte (black) in dye sensitized solar cell device under illumination of AM 1.5 G simulated solar light with $Co(bpy)_3^{2+/3+}$ redox mediator.

**Table S1.** Band assignments of Zn-perylene SURMOF-Bodipy/PMMA (Sample 1), Zn-perylene SURMOFs (Sample 2), Bodipy/PMMA (Sample 3), Bodipy powder (Sample 4), and perylene powder (Sample 5).

| Band position ($cm^{-1}$) | Sample | | | | | Band assignments |
|---|---|---|---|---|---|---|
| | 1 | 2 | 3 | 4 | 5 | |
| 1686 | | | | | yes | -COOH group of perylene compound |
| 1556 | yes | yes | | | | Zn-perylene MOFs thin film linkage |
| 1451 | yes | | yes | yes | | Lewis acid coordination between Zn-perylene + pyridine functionalized Bodipy |
| 965-975 | yes | | yes | | | 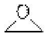 moiety of PMMA on MOFs thin film / Bodipy |
| 1435 | yes | | yes | | | Asymmetric bending of methyl group of PMMA |
| 1720-1755 | yes | | yes | | | [C=O] moiety / carbonyl group of PMMA |
| 1063 | yes | | yes | | | [C-O-C] moiety of PMMA |

**Table S2.** Performance characteristics of dye sensitized solar cell (DSSC) under AM1.5 irradiation.

| Nr. | Materials | FF (%) | *Jsc* [μA / $cm^{-2}$] | *Voc* [mV] | PCE (%) |
|---|---|---|---|---|---|
| **1** | **$TiO_2$ -Zn-perylene SURMOF** | 25 | 10.4 | 45 | 0.0419 |
| **2** | **$TiO_2$-Bodipy/ PMMA** | 23 | 10.2 | 33 | 0.0246 |
| **3** | **$TiO_2$ -Zn-perylene SURMOF Bodipy/PMMA** | 35 | 17.2 | 72 | 0.1185 |

## References


1. Xu, D.; Zhu, W.; An, Q.; Li, W.; Li, X.; Yang, H.; Yin, J.; Li, G. Clickable Inverse Opal: A Useful Platform for Fabrication of Stimuli-Responsive Photonic Materials. Chem Commun 2012, 48 (29), 3494-3496.
2. Wu, W.; Guo, H.; Wu, W.; Ji, S.; Zhao, J. Organic Triplet Sensitizer Library Derived from a Single Chromophore (Bodipy) with Long-Lived Triplet Excited State for Triplet–Triplet Annihilation Based Upconversion. The Journal of organic chemistry 2011, 76 (17), 7056-7064.
3. Liu, J.; Lukose, B.; Shekhah, O.; Arslan, H. K.; Weidler, P.; Gliemann, H.; Bräse, S.; Grosjean, S.; Godt, A.; Feng, X. A Novel Series of Isoreticular Metal Organic Frameworks: Realizing Metastable Structures by Liquid Phase Epitaxy. Sci Rep-Uk 2012, 2, 921.
4. Liu, J. X.; Zhou, W. C.; Liu, J. X.; Howard, I.; Kilibarda, G.; Schlabach, S.; Coupry, D.; Addicoat, M.; Yoneda, S.; Tsutsui, Y.; Sakurai, T.; Seki, S.; Wang, Z. B.; Lindemann, P.; Redel, E.; Heine, T.; Wöll, C. Photoinduced Charge-Carrier Generation in Epitaxial Mof Thin Films: High Efficiency as a Result of an Indirect Electronic Band Gap? Angew Chem Int Edit 2015, 54 (25), 7441-7445.


© 2018 by the authors. Submitted for possible open access publication under the terms and conditions of the Creative Commons Attribution (CC BY) license (http://creativecommons.org/licenses/by/4.0/).
